# Peripheral muscle oxygenation, pain, and disability indices in individuals with and without nonspecific neck pain, before and after myofascial reorganization^®^: A double-blind randomized controlled trial

**DOI:** 10.1371/journal.pone.0292114

**Published:** 2024-02-09

**Authors:** Mayane dos Santos Amorim, Larissa Sinhorim, Iramar Baptistella do Nascimento, Janaína Wagner, Francisco de Paula Lemos, Maria Elisa Duarte França, Robert Schleip, Anelise Sonza, Gilmar Moraes Santos

**Affiliations:** 1 College of Health Sciences and Sports at Santa Catarina State University (UDESC), Posture and Balance Laboratory (LAPEQ), Florianópolis, State of Santa Catarina, Brazil; 2 Associate Professorship of Conservative and Rehabilitative Orthopaedics, Department of Sport and Health Sciences, Technical University of Munich, Munich, Germany; 3 DIPLOMA Hochschule Bad Sooden-Allendorf, Bad Sooden-Allendorf, Germany; UFSCar: Universidade Federal de Sao Carlos, BRAZIL

## Abstract

To investigate whether myofascial reorganization^®^ in the trapezius muscle (MRT) improves peripheral muscle oxygenation and pain tolerance and decreases neck disability index (NDI) scores in individuals with and without nonspecific neck pain (NP) using a double-blind randomized controlled trial. Seventy-five subjects were equally and randomly assigned to three groups: the intervention groups (experimental [EG] and sham sSG]) and the control group (CG). Several inclusion criteria were applied to the intervention groups: male or female, aged 18–32 years, self-reported NP in the last 3 months without a defined cause; at least “soft” pain in session 1 of the NDI, and at least a score of 1 on the Visual Analogue Scale (VAS). The CG was required to have NDI and VAS scores of 0 at recruitment. Intervention: The EG underwent MRT for 10 min, once a week for 6 weeks. Patients with NP in the SG underwent classical massage for the same duration and frequency. Patients in the CG had no pain and underwent no intervention. Data collection was performed using the NDI Questionnaire, a pressure algometer for pain evaluation, and near-infrared spectroscopy for muscle oxygenation measurements. It was registered as NCT03882515 at ClinicalTrials.gov. The NDI score in both the EG (p<0.001) and SG (p<0.001) decreased after 6 weeks of intervention compared to the CG. The CG demonstrated a lower basal tissue saturation (TSI) index than the EG (p<0.001) and SG (p = 0.02). The EG demonstrated higher oxyhemoglobin values than the SG (p<0.001) and CG (p = 0.03). The CG had higher pain tolerance than the EG (p = 0.01) and SG (p<0.001) post-intervention. MRT increased trapezius muscle oxygenation after 6 weeks of intervention.

## Introduction

Nonspecific neck pain (NP) is a common disorder with a high prevalence in occupational sectors [1, 2]. It is defined as pain in the posterior and lateral aspect of the neck between the superior nuchal line and the spinous process of the first thoracic vertebra that does not present with neurological signs or specific pathologies, such as traumatic sprain or fracture, tumor, and infectious or inflammatory cervical spondylolysis, and has no or minor interference with daily living activities [3–8]. In most cases, a specific diagnosis cannot be made and NP is labeled as non-specific due to its multifactorial etiology [9–11]. The Global Burden of Disease Study stated that NP is the fourth largest physical complaint globally with regard to years lived with a disability [12]. The estimated 1-year incidence of NP has been reported to vary from 10.4–21.3% [13, 14]. NP may occur due to incorrect execution of shoulder or upper limb movements that depend on scapular stabilization during daily activity [15]. Among other factors, scapular stabilization requires control and activation of the rotator cuff and the lower and middle trapezius muscle (TM) fibers [16, 17]. The TM fibers are covered by a connective tissue called the fascia. The fascial system [18] has sufficient blood supply to perform its own functions and contains elastin and collagen fibers that provide plastic and elastic properties. However, the energy demands of the fascia during some activities may generate muscle overload, which may reduce local blood supply [19]. This decrease in tissue blood supply might lead to changes that limit or prevent myofascial tissues from sliding across each other, thereby generating myofascial adherence [20–24]. Several treatment options exist for fascial system dysfunction, including the physiotherapeutic technique of myofascial reorganization^®^ (MR). MR works through mechanotransduction, biotensegrity, and a combination of manual pressure with specific mechanical loads in the myofascial tissue [20, 25] MR involves the recognition of areas and paths of resistance and tension [20] to determine the duration, depth, and direction of the exerted pressure to tissue resistance [26–28]. MR can influence the mechanoreceptors inside the fascia, thus contributing to local fluid dynamics, reducing excessive muscle tension and capillary constriction, and increasing local blood flow [24]. These mechanoreceptors were discovered by Yahia et al. (1992) [29] and Stecco et al. (2013) [30] through histological examination of the fascial tissue. In addition, both author groups affirmed the likely participation of the fascial system in proprioception. A study on the acute effect of MR in individuals with NP was published in 2022 with promising results. The authors observed an increase in the tissue level of oxyhemoglobin [31]. As it only utilizes manual contact, MRT is a simple, non-invasive, non-pharmacological, and low-cost method that could be easily applied in clinical practice. Therefore, we aimed to investigate whether MRT improves peripheral muscle oxygenation as a primary objective and pain tolerance and neck disability index (NDI) scores in individuals with NP as secondary objective.

## Materials and methods

### Study design

We conducted a double-blind randomized controlled clinical trial. The participants were divided into three groups: (i) the experimental group (EG) who underwent MRT; (ii) the sham group (SG) who underwent classical massage (CM); and (iii) the control group (CG) who received no intervention. The EG and SG included participants with NP. The CG included participants without NP.

### Randomization

The participants were initially evaluated by a blinded trained investigator who, using the random number generator application, randomly assigned blocks for the EG and SG in parallel (1:1), using blinded allocation and intention-to-treat (ITT) analysis. During randomization, the participants with NP either underwent MRT or CM. The therapist did not participate in the selection and was blinded during the allocation. However, we could not blind the therapist regarding the groups. CM is a continuous superficial sliding technique, associated with benefits and wellbeing [32]. A CG of individuals without NP underwent an evaluation before and after 10 min, without any intervention.

### Population and sample

The study was approved by the Ethics and Research Committee on Human Beings of the Santa Catarina State University (protocol number: 2.630.855) on May 02, 2018. The study was registered as NCT03882515 at ClinicalTrials.gov on March 27, 2019. We confirm that all ongoing and related studies for this drug/intervention are registered. Each participant provided Informed written consent. Several inclusion criteria were applied to the intervention groups: participants of both sexes, aged > 18 years, with self-reported NP in the last 3 months without a defined cause. The NP should have specifically occurred in the TM during daily activities or work. Additionally, they should have had at least “soft” pain in session 1 of the NDI and at least a score of 1 on the Visual Analogue Scale (VAS). The CG was required to have scores of 0 for the NDI and VAS at recruitment. The CG were matched with the EG according to age, sex, and BMI, due to the relevance of these variables to the fascia. Several exclusion criteria were applied for both groups: neurological disease, history of trauma or surgery on the cervical spine, clinical diagnosis of disc herniation or nerve compressions, and previous physical therapy (3 months). Individuals who met the inclusion criteria and provided informed consent were invited to participate in the study. We informed participants about the possibility to withdraw their participation at any phase without penalty or constraints. We preserved the personal identification of all participants, due to the possibility of scientific dissemination of the results. Data were collected at the Santa Catarina State University. The participants were recruited using convenience sampling and allocated to the aforementioned groups. Data collection was carried out from August 2018 to July 2019.

### Sample calculation

**Fig 1:**
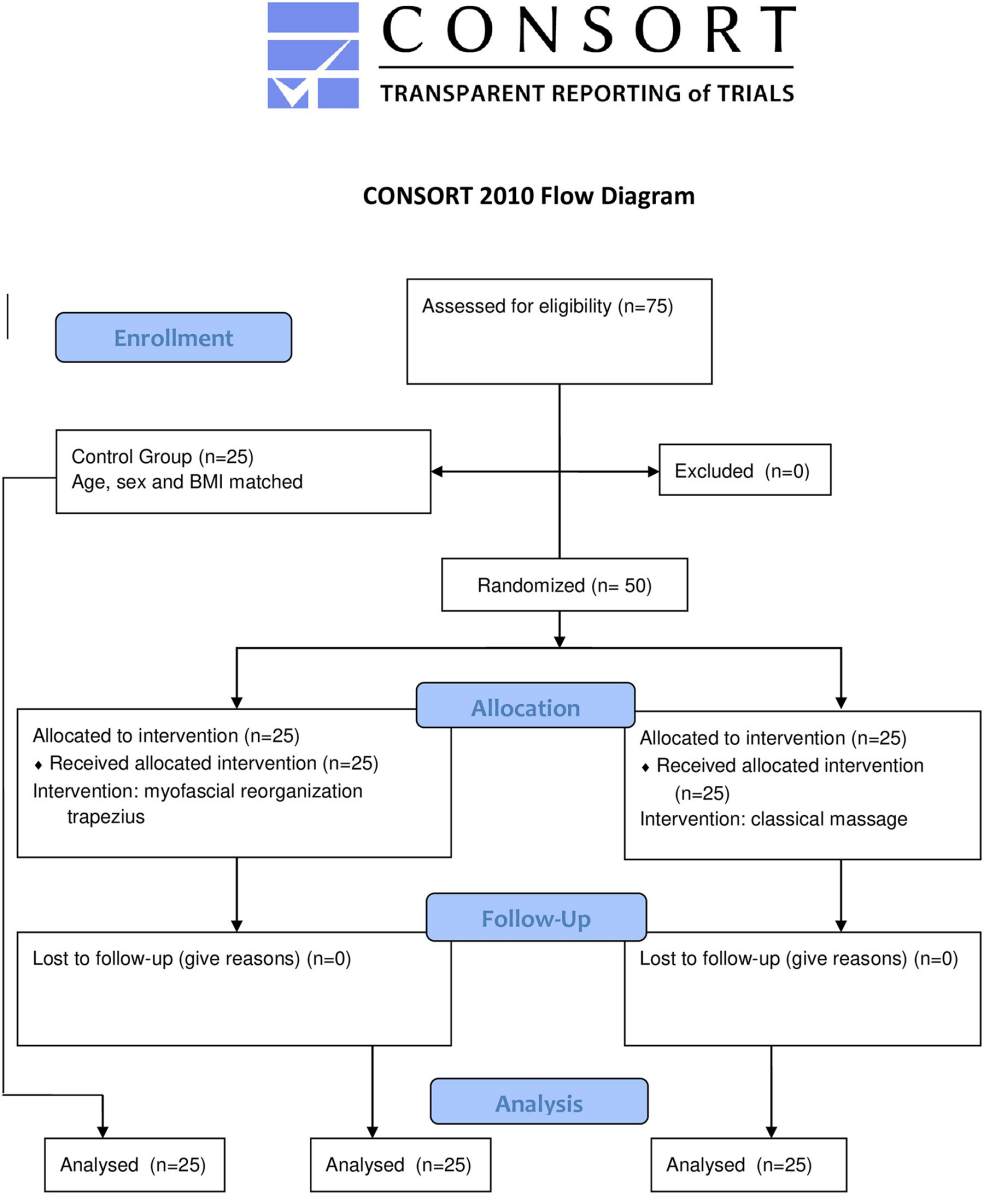
Flow diagram.

We estimated the number of participants for each group using the G*Power 3.0.10 software, with analysis of variance with repeated measures for intra-and inter-participants considering the three independent groups (EG, SG, and CG). A total sample of 54 participants (18 per group) provided a power of 95% and an effect size of 0.25 (Cohen’s d), once that there was no study to be based as expressed in Table 1. The type I error was fixed at the conventional level (5%). We defined the number of participants in each group using the key characteristics of the study, such as the study design, primary outcome, and variability–Fig 1 details the flow of patients.

**Table 1:** d-value in comparisons between groups.

Outcome Measure	Group/Time	Mean (Pre)	SD (Pre)	Mean (Pos)	SD (Pos)	Cohen’s d
**ΔO2Hb (μMol/l)**	Experimental	0.81	1.51	0.62	2.66	-0.621
**ΔHHb (μMol/l)**		0.07	0.77	-2.1	8.31	-0.103
**ΔtHb (μMol/l)**		0.71	2.48	0.69	1.89	0.187
**TSI (%)**		77.8	4.99	80.93	4.59	0.614
**ΔO2Hb (μMol/l)**	Sham	-0.16	1.45	-0.62	1.85	-0.778
**ΔHHb (μMol/l)**		0.62	1.06	-0.11	1.86	0.225
**ΔtHb (μMol/l)**		1.36	3.95	-0.47	2.87	-0.561
**TSI (%)**		77.72	5.79	78.5	3.75	0.219
**ΔO2Hb (μMol/l)**	Control	-0.19	1.33	0.45	1.27	-0.621
**ΔHHb (μMol/l)**		1.67	6.03	-0.05	0.65	-0.103
**ΔtHb (μMol/l)**		-0.16	1.97	-0.3	1.53	0.187
**TSI (%)**		75.36	4.98	75.54	5.16	0.614
**Pain**	Experimental	51.08	27.65	64.24	25.92	0.079
	Sham	42.78	22.69	45.58	27.91	-0.287
	Control	70.49	32.29	81.24	27.8	0.079
**Neck disability index**	Experimental	21.12	7.73	13.68	8.76	-1.234
	Sham	18.12	7.46	13.96	8.18	-0.751
	Control	5.48	5.58	5.08	6.98	-1.234

### Instrumentation

We used a near-infrared spectroscopy (NIRS; Artinis^®^, Portamon system, Netherlands), pressure algometer (JTech Commander Algometer^®^, USA), NDI questionnaire, [33] and VAS [34].

#### Anthropometric measurements

We measured the participants’ stature using a stadiometer (Standard Sanny—American Medical do Brasil Ltda, São Paulo, BRA) and body mass using a digital scale (Mettler-Toledo, Inc., Columbus, Ohio, USA^®^). Each participant’s BMI was calculated using these variables.

#### Near-infrared spectroscopy

We used NIRS (PortaMon^®^, Artinis, Netherlands) to measure peripheral muscle oxygenation. NIRS primarily depends on the relative transparency of a tissue to light and the light-absorbing characteristics of oxygenated hemoglobin. It continuously displays relative changes in hemoglobin concentration using different wavelengths (light-emitting diodes: 3×2 wavelengths). NIRS is wireless, non-invasive, accessible, and allows continuous measurement to be performed in a laboratory or even outdoors, without the need for special infrastructure [35].

#### Pressure algometer

We used a digital algometer (JTech Commander Algometer^®^, USA) for quantitative assessment of pain pressure thresholds (PPT). The pressure algometer provides a semi-objective, reliable, and efficient assessment for treatment planning and evolution monitoring [36].

#### Neck disability index

The NDI is a 10-item questionnaire designed to assess functional disability and pain around the cervical spine. It has been adapted and validated in the Brazilian Portuguese language [37]. Each item comprises six responses that are connected to a routine activity, except for question five, which is related to headaches. Responses are numbered from 0–5 and are associated with the degree of cervical pain and its interference with routine activities. The scores were calculated and converted into a percentage value and only the items answered were considered [33].

#### Visual Analog Scale

The VAS is a one-dimensional instrument used to assess subjective pain intensity. It comprises a line numbered from 0–10, wherein 0 corresponds to “no pain,” and 10 corresponds to the “the worst imaginable pain” [34].

### Outcome parameters

For each set of analyzed repetitions (3×3 matrix; three evaluations in three groups), several parameters were extracted: peripheral muscle oxygenation (oxyhemoglobin, O_2_Hb), deoxyhemoglobin (HHb), total hemoglobin (tHB), and the tissue saturation index (TSI) at the time of interest (before and immediately after the intervention). Other parameters, such as subjective pain intensity, pain tolerance, and NDI, were measured before the interventions.

### Data collection procedures

Participants were evaluated by a blinded evaluator who collected their personal (name, address, telephone, and age) and anthropometric data (stature and body mass). Thereafter, NDI and VAS scores were evaluated, followed by NIRS and algometer measurements in a separate intervention room. The rooms were maintained at a controlled temperature (24°C) during the measurements and interventions.

#### Primary outcomes

**Fig 2:**
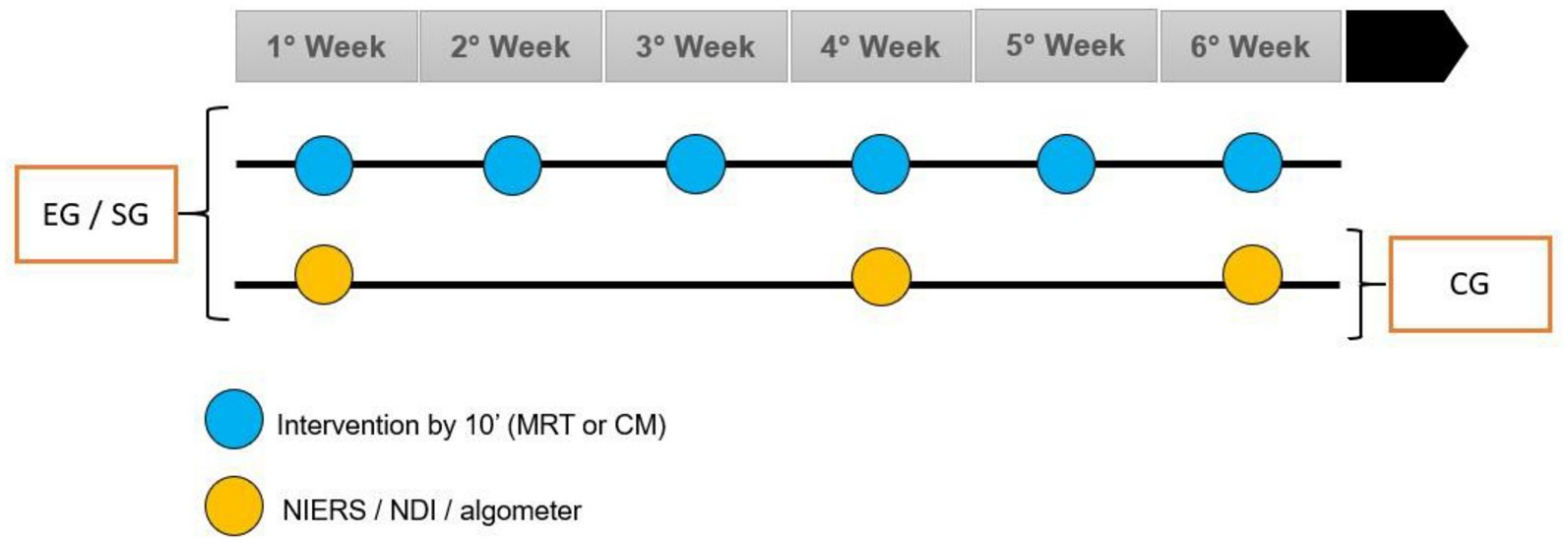
Data collection procedure scheme. Abbreviations: EG, experimental group; SG, sham group; CG, control group; MRT, myofascial reorganization^®^ trapezius muscle; CM, classical massage; NIRS, near-infrared spectroscopy; NDI, neck disability index.

Muscle oxygenation was measured before and after the intervention, or after 10 min rest for the CG. Skin was cleaned with alcohol, following which the device was positioned bilaterally in the middle fibers of the TM, based on the Surface ElectroMyoGraphy for the Non-Invasive Assessment of Muscles (SENIAM) recommendations) in contact with the skin. Those with hairy skin underwent trichotomy using disposable and individual blades. The equipment was covered with a black cloth to avoid light penetration. The acquisition frequency was 10 Hz. The data collection procedure comprised six steps of intervention/evaluation (Fig 2).

#### Secondary outcomes

To assess pain tolerance, the participants were seated on a backless chair and an algometer was positioned bilaterally in the middle fibers of the TM at the T3 level, according to SENIAM recommendations [38]. The evaluator applied pressure perpendicular to the skin until the pressure pain tolerance was acknowledged by the participant [39]. The procedure was repeated twice on each side of the TM.

The EG and SG underwent the MRT and CM, respectively, once a week for 10 min, for 6 weeks. The CG underwent three assessments during the 6 week period (weeks 1, 4, and 6). Following evaluation using NIRS, NDI, algometer, and VAS, the participants were requested to rest for 10 min, followed by repeat measurements. In cases of absences, the intervention was postponed for a week as participants had to receive six interventions to conclude the study.

### Interventions

**Fig 3:**
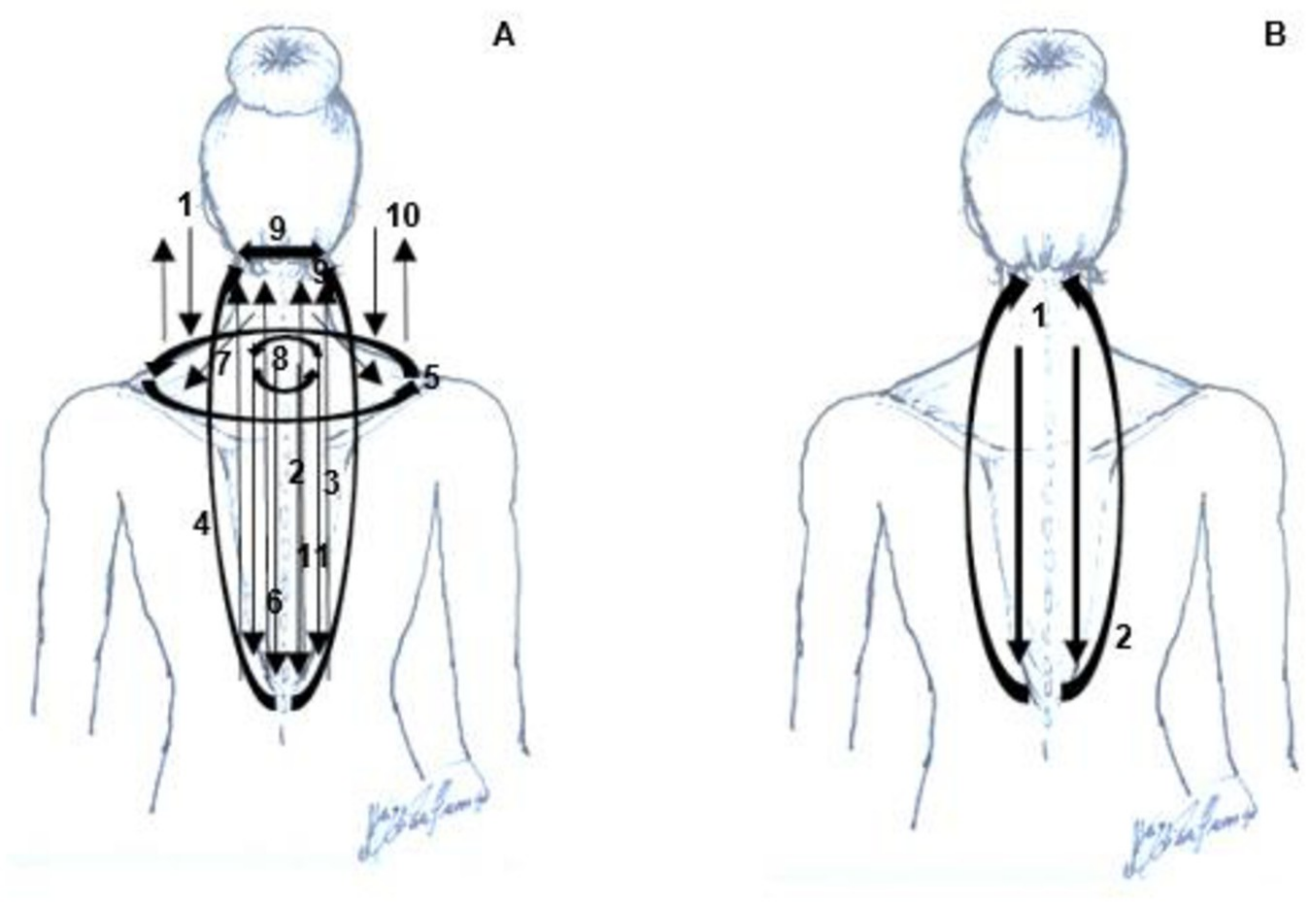
A schematic of the protocols performed: (A) MRT and (B) CM techniques (SINHORIM, et al., 2019). (A) (1): Participants began in a side position. Pressure was applied with a closed fist and movement in shear in the medium fibers of the TM. (2–9): Participants were moved to a prone position. (2–3) Sliding along the TM to the base of the skull, (4) Sliding of the T12 level around the lateral edge of the scapula to the axilla, (5) Sliding forming a canoe from one acronym to another, passing above and below the level of T1, (6) Compression in the caudal direction from the upper to the lower TM in the thenar region, (7) Sliding of the proximal and distal insertion of TM medial fibers, (8) Sliding bypassing the T1 spinous process, and (9) Sliding transversely at the upper insertion of the upper TM fibers. (10) Participants were moved to the supine position and tweezer traction of the TM fibers was performed. (11) Participants were seated with their hands in a fist, and continuous clamping and shear on the TM was performed. (B) (1) Participants were placed in a prone position. Continuous superficial displacement at the lateral edges of the TM followed the directional movement of the lower, medium, and upper fibers. (2) Caudal–cranial return of the technique. Abbreviations: MRT, myofascial reorganization^®^ trapezius; CM, classical massage; TM, trapezius muscle.

The protocols were performed by the same trained physiotherapist and each session lasted 10 min. The MRT followed the protocol published by Sinhorim et al. (2019) [20] and Amorim et al. (2022) [31]. This manipulation technique emphasizes specific mechanical loads directed to the fascial tissue. The CM technique was based on the studies by Domenico and Wood (1998) and Kong et al. (2013) [32, 40]. The intervention room had a stretcher, chair, and bathroom for the convenience of the participants. Fig 3 provides a schematic representation of the protocol.

### Data processing and statistical analyses

We extracted the muscle tissue oxygenation data (HbO_2_, HHb, tHB, and TSI [%] concentrations) using dedicated software (Oxysoft^®^, Artinis, Netherlands). The deltas of variations in the obtained concentrations were calculated, and the average tissue oxygen saturation was calculated before and immediately after the intervention. The PPT data were obtained from the equipment and extracted from the mean of two bilateral measurements. We calculated the NDI scores for each participant and the VAS was registered before the interventions or after 10 min rest for the CG. The data were initially tabulated in Microsoft Excel using the Windows 10 operating system. Then, they were converted to the Statistical Package for the Social Sciences software (SPSS) version 20.0. To verify the normal distribution of the sample, the Kormogorov-Smirnov test was applied. Descriptive statistics were used to characterize the participants through means and standard deviations. To analyze whether MRT mediated changes in muscle oxygenation (HbO2, HHb, tHB, and TSI), a statistical model was constructed based on inter- and intra-group comparisons using a general linear variance analysis (repeated measures 3×3). This helped detect differences between the mentioned variables across different periods. The level of significance was set at p≤0.05. The results were reported in accordance with CONSORT. In all comparisons, a primary ITT analysis including all randomized participants was performed. For ITT analysis, missing data were filled in using simple imputation, being replaced by the last value collected from the participant for the variable in question.

## Results

This study included 56 females and 19 males aged 18–39 years, who were divided into three groups, (EG, SG, and CG). A total of 300 interventions were performed during the 6 week intervention period across the EG (MRT) and SG (CM). Table 2 summarizes the sample characteristics.

**Table 2:** Anthropometric characterization by group.

	EG	SG	CG	P value
Gender (male and female)	7 and 18	5 and 20	7 and 18	
Age (years)	22.60±3.85 male (23.71±1.93) female (22.17±0.79)	22.56±3.75 male (24.20±2.49) female (22.15±0.71)	22.64±3.80 male (23.71±1;92) female (22.22±0.76)	0.997
Mass (kg)	67.00±14.78 male (77.62±5.31) female (62.86±3.10)	64.60±10.68 male (73.10±5.10) female (62.48±2.15)	65.20±11.52 male (79.86±2.91) female (59.50±1.55)	0.779
Stature (m)	1.67±0.08 male (1.75±0.02) female (1.64±0.01)	1.64±0.06 male (1.73±0.01) female (1.62±0.01)	1.67±0.08 male (1.78±0.02) female (1.62±0.01)	0.360
BMI (kg/m^2^)	23.84±4.78 male (25.56±2.38) female (23.21±0.96)	23.80±3.15 male (24.26±1.25) female (23.68±0.73)	23.14±2.50 male (24.95±0.79) female (22.43±0.55)	0.731

Data are expressed as mean±standard deviation. EG, experimental group; SG, sham group; CG, control group; kg, kilogram; m, meters; BMI, body mass index.

Table 3 depicts the TM oxygenation values, qualitative and quantitative pain results, and the NDI scores for the groups.

**Table 3:** Peripheral muscle oxygenation, pain, and neck disability index scores across the studied groups (EG: 25; SG: 25; and CG: 25) during the 1^st^, 4^th^, and 6^th^ weeks of evaluation.

Variables	1^st^ Week			4^th^ Week			6^th^ Week		
	EG	SG	CG	EG	SG	CG	EG	SG	CG
**ΔO**_**2**_ **Hb (μMol/l)**	0.81±1.51	-0.16±1.45	-0.19±1.33	1.28±2.36	-1.18±1.76	0.82±2.97	0.62±2.66	-0.62±1.85	0.45±1,27^‡^
**ΔHHb (μMol/l)**	0.07±0.77	0.62±1.06	1.67±6.03	0.008±1.15	0.49±1.12	0.26±1.04	-2.10±8.31	-0.11±1.86	-0.05±0.65
**ΔtHb (μMol/l)**	0.71±2.48	1.36±3.95	-0.16±1.97	0.87±3.30	-0.65±2.14	0.61±3.05	0.69±1.89	-0.47±2.87	-0.30±1.53
**TSI (%)**	77.80±4.99	77.72±5.79	75.36±4.98	79.90±7.29	79.26±5.35	75.62±4.48	80.93±4.59*	78.50±3.75	75.54±5.16
**Pain (Kgf)**	51.08±27.65	42.78±22.69	70.49±32.29	53.15±22.78	42.07±28.18	78.55±29.93	64.24±25.92	45.58±27.91	81.24±27.80
**NDI (%)**	21.12±7.73	18.12±7.46	5.48±5.58	17.88±8.81	17.36±7.68	5.76±6.19	13.68±8.76^†^	13.96±8.18^†^	5.08±6.98

Data are expressed as mean ± standard deviation; EG: experimental group, SG: sham group, CG: control group, ΔO_2_Hb: oxyhemoglobin, ΔHHb: deoxyhemoglobin, ΔtHb: total hemoglobin, TSI: tissue saturation index, μMol / l: micromol per liter, %: percent, Kgf: kilogram force, NDI: Neck Disability Index.

*Statistically significant EG values compared to the 1^st^ week (p<0.001) for TSI;

^†^statistically significant values of EG (p<0.001) and SG (p<0.001) for NDI (ANOVA test of repeated measures); and

^‡^ statistically significant EG values when compared to the SG (p<0.001) and CG (p = 0.03) for O_2_Hb.

### NDI findings

We observed an interaction (weeks × groups) between the NDI results (F(1, 272) = 7.310; p<0.001). Following 6 weeks of treatment, we observed a significant reduction in the functional disability scores in the EG (p<0.001) and SG (p<0.001) compared to the CG (Table 3). After six interventions, the EG, SG, and CG demonstrated score decreases of 35.22%, 22.95%, and 8.96%, respectively.

### Pain tolerance

There was an increase in PPT (F(1, 172) = 18.083; p<0.001) from week 1 to week 6 (p<0.001) and from week 4 to week 6 (p<0.001; Table 3). The differences between groups were statistically significant (F(1, 172) = 10.553; p<0.001). Regardless of the week of evaluation, the CG demonstrated greater PPT than the EG (p = 0.01) and SG (p<0.001). At the end of the treatment, the EG, SG, and CG demonstrated 25.8%, 6.5%, and 15.3% increases in PPT respectively. Therefore, the percentage increase in PPT was greater in the EG.

### Peripheral muscle oxygenation

The TSI findings revealed a significant difference between the groups (F(1, 272) = 7.206; p<0.001). The CG demonstrated a lower baseline TSI than the EG (p<0.001) and SG (p = 0.02). The percentage increases in the EG, SG, and CG were 4.02%, 1%, and 0.23%, respectively. The EG presented higher (F(2, 172) = 11.699; p<0.001) HbO2 values than the SG (p<0.001) and CG (p = 0.03) and demonstrated a positive percentage variation (post-treatment − pre-treatment) across the treatment duration. This behavior differed from that of the SG and CG, which occasionally demonstrated negative variation. Other variables (ΔHHb and ΔtHb) related to peripheral muscle oxygenation did not demonstrate significant differences between the groups and weeks.

## Discussion

Our findings revealed that MRT modified pain tolerance, peripheral muscle oxygenation, and NDI among individuals with and without NP at three time points (before and after the 1^st^, 4^th^, and 6^th^ intervention), thereby confirming our hypothesis. This is the first study to use this methodological proposal.

The EG demonstrated an increase in TSI relative to the CG, in addition to a higher percentage increase (4.02%) in tissue saturation than the SG and CG. Moreover, the EG had a higher PPT increase (25.8%) than the SG (6.5%) and CG (15.3%). The EG and SG presented lower functional disability scores, indicating less disability at the end of the treatment. The EG exhibited a a greater reduction in functional disability scores (35.22%) than the SG (22.95%). In a recently published study, 50 participants (female: 36, male: 14) with and without nonspecific NP were assigned to either the EG (25 participants with NP), who underwent MRT or the CG (without NP) who did not undergo the intervention. TM oxygenation was measured using NIRS before and after a single intervention. All participants were tested and re-tested after 10 min. Results indicated that after 10 min of MRT, the level of oxygenoglobin in the middle fibers of the trapezius increased (0.72 ± 1.47 vs. −0.14 ± 1.33 mmol/dL, p = 0.01) [40].

These findings corroborate the results of a previous study that comprised 59 individuals with NP who underwent 10 treatment sessions across 4 weeks, each lasting 15 min. The treatment consisted of analgesic superficial thermotherapy and transcutaneous electrical stimulation (50 min) with either manipulation therapy or MRT in the sternocleidomastoid, supra, and infrahyoid muscles and posterior rectus head muscle [41]. The researchers observed significant pain reduction in both groups. This reduction was greater in the group that received MRT (22.1–16.0) than the group that underwent manipulation therapy (24.5–20.0). However, this difference was not significant. Similarly, we observed a significant difference in PPT between those who underwent MRT and CM at the end of the treatment.

Our study demonstrated increased PPT in the group that received MRT after six interventions. Our findings were similar to that of another study that allocated 41 participants with NP [42] to two groups: those who received MRT (five 45-min sessions) and those who underwent ultrasound therapy, transcutaneous electrical nerve stimulation, and massage (10 sessions across 2 weeks). The authors concluded that MRT was better than a multimodal physiotherapy program for pain improvement.

Several chronic and persistent dysfunctions are supposedly related to transmission of prolonged nociceptive signals to peripheral myofascial tissues, which sensitize the central nervous system and increase its excitability [43]. Pro-inflammatory processes [44] cause nociceptive stimuli [45] and trigger momentaneous adaptations in endogenous pain modulation [46]. At the end of 6 weeks, we observed a greater percentage increase in PPT in the EG (25.8%) than in the SG (6.5%). This could have been caused by the MRT-promotedremodeling through mechanotransduction, which is a force applied to the molecular mechanical system that triggers intracellular biochemical exchanges [41], thus, leading to analgesia.

Muscle pain can occur due to intramuscular hypoperfusion, [47] which involves the impairment of muscle tissue microcirculation, resulting in nociceptive pain. Disturbances in muscle microcirculation can produce increased nociceptive neuronal activity by sensitizing the regional muscle nociceptors. In our study, the symptomatic participants (EG and SG) primarily comprised sedentary university students who spent long hours in a sitting position, overloading the TM and, thus, possibly constantly stimulating nociception. This corresponds to the findings of Cagnie et al. [48], who analyzed workers who stayed at the computer for at least 4 h per day.

Performing TM-related movements can decrease muscle oxygenation, as reported by Elcadi et al., [49] who investigated oxygenation in the TM during a 5 min low-intensity contraction (10%). The authors observed a significant decrease in oxygenation during contraction. Other researchers [50, 51] have investigated the TSI in the middle third of the upper TM using NIRS and observed a decrease in muscle oxygenation during repetitive work, from 83.1% to 77.4%. In our study, following MRT, a higher percentage increase in TSI as seen in the EG (4.02%) than the SG and CG (1% and 0.23%, respectively) after six interventions. Our results corroborate those of Schah et al,. [52] who reported an oxygenation increase in the lumbar paravertebral muscles of asymptomatic individuals 30 min post-MRT implementation.

According to some studies [53–55], individuals with myofascial dysfunction experience intramuscular pressure and mechanical compression of the vessels that supply the TM. These changes can be attributed to the presence of myofascial adhesions [56] that decrease the blood supply to the muscle. This alteration in perfusion generates a consequent inefficiency of aerobic metabolism (decreased oxygenation) [57], which results in use of anaerobic metabolism, which creates a greater propensity for muscle fatigue [58].

MRT facilitates readjustment of myofascial structures, thereby increasing muscle oxygenation immediately following its application. Our results supported this premise, as MRT significantly altered the TSI and HbO_2_ and, consequently, peripheral muscle oxygenation.

### Study limitations

The present study has several limitations that should be acknowledged when interpreting the findings. Firstly, it is important to acknowledge that the aforementioned limitations arise from the lack of complete control over external variables, such as medication use, frequency of physical activity, and stress levels. These factors, which may have influenced the outcomes, were particularly relevant given that the sample predominantly consisted of undergraduate students. Moreover, the study did not assess the menstrual cycle of female participants or their caffeine intake on the day of the intervention.

Additionally, it is worth noting that the reassessment was conducted immediately after the final intervention, thereby limiting the availability of additional data points for analysis. To gain valuable insights into the long-term effects of the intervention, it is recommended to consider a more extended follow-up period or conduct multiple reassessments at different time points.

Furthermore, while the study revealed a statistically significant difference in NDI (Neck Disability Index) between the groups over multiple weeks, and an acceptable effect size was observed, caution is advised in interpreting these results. The absence of the recommended 8.4-point difference in pain intensity between the groups, as suggested by Jorritsma et al. (2012) [59], indicates a potential limitation in the clinical significance of the observed results.

Lastly, it is important to consider the limited feasibility of establishing the minimum clinically important difference for the Visual Analog Scale (VAS) within the specific context of the Brazilian population. The lack of established guidelines for the VAS in this context introduces uncertainty and hinders the comprehensive interpretation of pain intensity outcomes.

These identified limitations underscore the need for further investigation and consideration when assessing the implications of the study. Future research endeavors should aim to address these limitations by employing larger sample sizes, incorporating culturally specific guidelines for outcome measures, and exploring alternative methodologies to determine clinically significant differences in pain intensity.

## Conclusion

This study provides initial evidence that a 6-week regimen of weekly 10-minute myofascial reorganization^®^ (MR) sessions may improve trapezius muscle tissue oxygenation and peripheral blood flow among patients with neck pain. While the experimental group (EG) and sham group (SG) demonstrated statistically significant improvements in trapezius muscle tissue saturation index (TSI) compared to the control group (CG), the between-group differences in self-reported pain and disability scores did not exceed minimal clinically important difference (MCID) thresholds. This indicates that despite measurable physiological changes, the clinical meaningfulness of MR in altering patient-centered outcomes remains unclear in this sample. Future studies should investigate optimal MR parameters (frequency, duration) and include longer follow-up periods to assess sustainability of effects. Combining MR with other evidence-based interventions may also be worthwhile to determine any additive or synergistic benefits for individuals with neck pain.

In conclusion, this preliminary study showed that MR can induce measurable improvements in tissue perfusion in patients with neck pain. However, future randomized controlled trials are needed to confirm the efficacy and generalizability of these findings.

## Supporting information

S1 Checklist CONSORT 2010 checklist of information to include when reporting a randomised trial*. (DOC)

S1 File (XLSX)
